# Respiratory Bacterial Culture Sampling in Expectorating and Non-expectorating Patients With Cystic Fibrosis

**DOI:** 10.3389/fped.2018.00403

**Published:** 2018-12-18

**Authors:** Hanneke Eyns, Denis Piérard, Elke De Wachter, Leo Eeckhout, Peter Vaes, Anne Malfroot

**Affiliations:** ^1^Department of Pediatrics, Pediatric Pulmonology and Pediatric Infectious Diseases, Cystic Fibrosis Clinic, Universitair Ziekenhuis Brussel, Vrije Universiteit Brussel, Brussels, Belgium; ^2^Department of Physical Medicine and Physiotherapy, Universitair Ziekenhuis Brussel, Vrije Universiteit Brussel, Brussels, Belgium; ^3^Department of Physiotherapy, Human Physiology and Anatomy, Faculty of Physical Education and Physiotherapy, Vrije Universiteit Brussel, Brussels, Belgium; ^4^Department of Microbiology and Infection Control, Universitair Ziekenhuis Brussel, Vrije Universiteit Brussel, Brussels, Belgium

**Keywords:** cystic fibrosis, respiratory samples, cough swab, sputum, bronchoalveolar lavage (BAL)

## Abstract

**Purpose:** Different respiratory sampling methods exist to identify lower airway pathogens in patients with cystic fibrosis (CF), of which bronchoalveolar lavage (BAL), and expectorated sputum are considered the “gold standard.” Because BAL cannot be repeated limitless, the diagnosis of lower respiratory tract infections in non-expectorating patients is challenging. Other sampling techniques are nasal swab, cough swab, and induced sputum. The purpose of this study (NCT02363764) was to compare concordance between the microbiological yield of nasal swab, cough swab, and expectorated sputum in expectorating patients; nasal swab, cough swab, and induced sputum in non-expectorating patients; nasal swab, cough swab, induced sputum, and BAL in patients requiring bronchoscopy (“BAL-group”); and to determine the clinical value of cough swab in non-expectorating patients with CF.

**Methods:** Microbiological yield detected by these different sampling techniques was compared between and within 105 expectorating patients, 30 non-expectorating patients and BAL-group (*n* = 39) in a single CF clinic. Specificity, sensitivity, positive (PPV), and negative (NPV) predictive values were calculated.

**Results:** Overall low sensitivity (6.3–58.0%) and wide-ranging predictive values (0.0–100.0%) indicated that nasal swab was not appropriate to detect lower airway pathogens [*Pseudomonas aeruginosa* (*Pa*), *Staphylococcus aureus* (*Sa*), and *Haemophilus influenzae* (*Hi*)] in all three patient groups. Microbiological yield, specificity, sensitivity, PPV, and NPV of cough swab and induced sputum were largely similar in non-expectorating patients and in BAL-group (except sensitivity (0.0%) of induced sputum for *Hi* in BAL-group). Calculations for *Pa* and *Hi* could not be performed for non-expectorating patients because of low prevalence (*n* = 2 and *n* = 3, respectively). In expectorating patients, concordance was found between cough swab and expectorated sputum, except for *Hi* (sensitivity of 40.0%).

**Conclusion:** Our findings suggest that cough swab might be helpful in detecting the presence of some typical CF pathogens in the lower airways of clinically stable patients with CF. However, in symptomatic patients, who are unable to expectorate and who have a negative cough swab and induced sample, BAL should be performed as it currently remains the “gold standard.”

## Introduction

The most important site of disease and the predominant cause of both morbidity and mortality in cystic fibrosis (CF) is the respiratory tract. Chronic infection results in a prolonged inflammatory response, which is believed to cause respiratory tissue injury leading to progressive loss of pulmonary function (1, 2). While *Haemophilus influenzae* (*Hi*) and *Staphylococcus aureus* (*Sa*) may predominate early in life, ~20% of children with CF aged 2–5 years and 55–75% of adults with CF are chronically infected with *Pseudomonas aeruginosa* (*Pa*) (3). Certain bacteria, such as *Pa*, are associated with a worse clinical outcome than others, but can be completely eradicated if identified early and treated promptly. There is sufficient evidence that eradication of early infection and prevention of chronic infection is associated with clinical benefit (1, 3, 4). Therefore, accurate identification of lower respiratory tract pathogens is crucial in the management of CF and is recommended to be performed at least every 3 months using bacterial culture sampling (2, 5). Samples can be obtained through different methods of which bronchoalveolar lavage (BAL) is considered to be the “gold standard” method (6). Because BAL is an invasive method which cannot be repeated limitless, spontaneously expectorated sputum sample is accepted as second best (7). This, however, implies that the diagnosis of lower respiratory tract infections in the non-expectorating patient with CF can be challenging. Other methods (Table 1), such as induced sputum, cough swabs, throat swabs, and nasal swabs, have been developed to obtain bacterial cultures in these non-expectorating patients (7–12). Studies investigating these different sampling methods, however, reported conflicting results (7–12).

**Table 1:** Definition of different sampling methods for bacterial cultures (7–12).

**Method**	**Definition**
Bronchoalveolar lavage	Fluid squirted into and recollected from the lungs during bronchoscopy
Cough swab	Swab placed into the posterior pharynx, without direct contact with the oropharyngeal mucosa, and asking the patient to cough
Induced cough swab or Induced sputum sample	Cough swab or sputum sample obtained following inhalation of hypertonic saline
Nasal suctioning	Suctioning of the nasal cavity
Nasal swab	Swab of the nasopharyngeal wall
Oropharyngeal suctioning	Suctioning of the oropharynx
Oropharyngeal swab or Throat swab	Swab of the posterior oropharyngeal wall
Expectorated sputum sample	Spontaneous expectoration of sputum

Despite the available literature, questions about sampling methods used for respiratory bacterial cultures in the non-expectorating patient with CF remain unanswered. Is the microbiological yield of nasal swab, cough swab, and expectorated or induced sputum the same for each sampling method, and in expectorating vs. non-expectorating patients? Are microbiological results of these techniques as sensitive and as specific as those of BAL? What is the clinical value of cough swab for the identification of bacterial pathogens in the lower airways of the non-expectorating patient with CF? The aim of this study is to answer these questions by comparing results for prevalence, sensitivity, specificity, positive, and negative predictive value of nasal swab, cough swab, and expectorated or induced sputum and BAL.

## Methods

### Participants

Confirmed diagnosis of CF by sweat test and/or two identified CF-causing CFTR-mutations was mandatory for eligibility. All patients attending the Cystic Fibrosis Clinic of the Universitair Ziekenhuis (UZ) Brussel for their annual assessment or for a clinically indicated bronchoscopy between January 2015 and August 2016, were informed of the study by the specialized CF physiotherapist and invited to participate. Clinical indications for bronchoscopy were: persistent infection with clinical symptoms not improving despite antibiotic treatment and/or radiographic abnormalities such as newly acquired atelectasis not resolving with chest physiotherapy, anatomical abnormalities, external compression of the bronchi, etc.

The study protocol was approved by the Ethics Committee (O.G.16) of the UZ Brussel (B.U.N. 143201422976) and has been registered at ClinicalTrials.gov (NCT02363764). Parents, legal guardians, or of-age subjects provided written informed consent in accordance with the declaration of Helsinki prior to enrolment in this prospective study. Patients under the age of 18 were asked to provide assent consent.

### Sampling Procedures

Baseline demographic data (Table 2) including age, gender, body mass index (BMI), lung function, and CF-causing CFTR-mutation were obtained prior to sampling.

**Table 2:** Baseline demographic data.

	**Expectorating patients**	**Non-expectorating patients**	**BAL-group**	**Sign**.
*N*	105	30	39	
Children (018 years) (*n*, %)	42 (40.0%)	29 (96.7%)	29 (74.4%)	
Adults (≥ 18 years) (*n*, %)	63 (60.0%)	1 (3.3%)	10 (25.6%)	
Gender, n male (%)	58 (56.3%)	18 (61.1%)	13 (33.3%)	
Age (mean, ± *SD*)	23.9 (± 11.6)	9.2 (± 4.8)	13.4 (±10.3)	***p*** **=** **0.000**
BMI (mean, ± *SD*)	20.2 (± 3.4)	16.7 (± 2.8)	17.4 (± 3.3)	***p*** **=** **0.000**
FEV_1_ (%pred) (mean, ± *SD*)	70.8 (± 24.8)	94.4 (± 13.9)	63.1 (± 25.5)	***p*** **=** **0.000**
FVC (%pred) (mean, ± *SD*)	86.0 (± 18.0)	95.9 (± 12.1)	77.7 (± 23.4)	***p*** **=** **0.004**
**CF mutation**				
Homozygous F508del	49	8	26	
Other mutations^a^	56	22	13	

BAL-group, patients requiring clinically indicated bronchoscopy; Sign., significance level; BMI, Body Mass Index; FEV_1_, Forced Expiratory Volume in 1 second; FVC, Forced Vital Capacity;

a *Heterozygous F508del or Other/Other. Bold values indicates significant difference between the three groups*.

Patients attending the CF Clinic for their annual assessment were divided into two groups depending on their ability to expectorate sputum on demand. From expectorating patients, a nasal swab, cough swab and spontaneously expectorated sputum, were obtained. From non-expectorating patients a nasal swab, cough swab, and induced sputum/cough swab were obtained. Patients attending the CF Clinic for a clinically indicated bronchoscopy (BAL-group) provided a nasal swab, cough swab, induced sample (all obtained by the CF physiotherapist), and BAL (obtained by the pulmonologist).

To avoid microbiological contamination of the samples, sequential samples were collected as described below:

- *Nasal swab*: The patient was seated on a chair or on the lap of his parent with the head tilted slightly backwards. The physiotherapist inserted the swab (regular size nylon flocked swab, eSwab™, Copan Diagnostics Inc., Brescia, Italy) gently through the left and right nostril, respectively, into the nasopharynx and swirled it back and forth. The swab was then removed from the nose and placed into the accompanying tube filled with 1 ml of liquid Amies to send to the laboratory.
- *Cough swab*: After mouth-rinsing with water to avoid contamination from the nasopharynx the patient was assisted by the physiotherapist during a 10-min airway clearance session [autogenic drainage (AD)]. Subsequently, a swab was placed into the oropharynx without touching the pharyngeal mucosa and the patient was instructed to cough onto the swab (regular size nylon flocked swab, eSwab™, Copan Diagnostics Inc., Brescia, Italy). If a participant was too young to understand the instruction, a cough reflex was used to make the subject cough. The swab was then removed from the oropharynx and placed into the accompanying tube filled with 1 ml of liquid Amies to send to the laboratory.
- *Expectorated sputum*: The patient rinsed his mouth a second time and was asked to cough and expectorate his sputum into a sterile container.
- *Induced sputum/cough swab*: If the patient was unable to expectorate sputum on demand after obtaining the cough swab he was asked to inhale 4 ml of hypertonic saline (“HS”, NaCl 6%) nebulized by the patient's own device (Pari Boy (SX) or eFlow Rapid, PARI GmbH, Starnberg, Germany) after premedication (10-min delay) with salbutamol (100 μg via metered dose inhaler + spacer). The subject was then encouraged to cough and expectorate his sputum into a sterile container. If the patient was still unable to expectorate sputum a second cough swab was obtained by the physiotherapist following the method as explained previously but without another airway clearance session.

Nasal swab, cough swab, and induced sputum/cough swab were obtained within the hour prior to bronchoscopy, but before sedation. All subjects were monitored transcutaneous for oxygen saturation and heart rate (Nellcor™ N-600X with OxiMax™, Medtronic, Minneapolis, USA) and received supplementary oxygen as needed. The flexible fiber bronchoscope (Olympus BS XP160S 2.8, BS 3C160 3.8, 1T180 6.4, Olympus Europa SE&CO. KG, Hamburg, Germany) was always (both in children and adults) inserted orally to avoid nasal contamination. In addition, use of the suction channel was avoided until the tip of the bronchoscope passed the carina. A BAL-sample was collected by the pulmonologist during bronchoscopy as follows: aliquots of 0.5 ml/kg per lobe (total max. = 100 ml) of isotonic saline warmed to body temperature (36.5°C) were instilled and then recollected by mechanical suction in an aseptic disposable for examination. Pediatric patients were sedated according to age and weight. Children under 8 months and/or 6 kg received atropine intrarectally and tetracaine 1% in NaCl 0.9% in the nose (2 ml) and pharynx (max. 6 ml). Children over 8 months and 6 kg were sedated with 0.6 ml/kg midazolam (5 mg/ml; max. 1.5 ml), 0.6 ml/kg atropine (0.25 mg/ml; max. 1 ml), 0.02 ml/kg tramadol hydrochloride (50 mg/ml, max. 0.6 ml), and tetracaine 1% in NaCl 0.9% in the nose (2 ml) and pharynx (max. 6 ml). In adult patients only topical anesthesia was applied to the pharynx (lidocaine hydrochloride 10%; three sprays with a 5-min interval) and vocal cords (0.8 ml lidocaine hydrochloride 2%).

### Microbiological Analyses

All samples were sent to the laboratory within 1 h, stored at 4°C and processed within 24 h (13). Sample specimens were inoculated onto horse blood agar supplemented with X and V factors and vancomycin, bacitracin and clindamycin, mannitol salt agar, MacConkey agar, and *Burkholderia cepacia* selective agar for isolation of organisms associated with CF lung disease: *Pa, Sa, Hi, Burkholderia species, Streptococcus pneumoniae, Enterobacteriaceae*, and other non-glucose fermenting Gram negative organisms. Isolated colonies were identified by MALDI-TOF mass spectrometry and antibiotic susceptibility testing was performed by disc diffusion.

### Data Analyses

Based on the study by Equi et al. who obtained CS from 161 patients and compared these to expectorated sputum in a subgroup of 30 patients, we calculated that sample size had to be at least 114 patients for the initial sampling study, if a 5% margin of error and 95% confidence intervals were considered (10). To take a 15% loss of data into account (e.g., patients denying participation, patients not tolerating HS-inhalation, loss of samples, etc.), sample size was increased to 134 subjects.

Continuous variables (age, BMI, FEV_1_%pred, FVC) were compared using ANOVA and *post-hoc* Bonferroni. Differences between groups (expectorating patients, non-expectorating patients and BAL-group) and within groups (each sample method) were calculated from 3 × 2- or 4 × 2-tables. Bonferroni-correction was applied where needed. Sensitivity, specificity and positive (PPV) and negative (NPV) predictive values, with corresponding 95% binomial confidence intervals (95%CI) were calculated from 2 × 2-tables if *n* ≥ 5 (9, 14). Expectorated sputum, cough swab and BAL were used as the reference sample in the expectorating patients, non-expectorating patients and BAL-group, respectively.

Statistical analyses were performed using SPSS Statistical Software Version 25 for Windows (IBM, Armonk, NY, USA). For all analyses, unless stated otherwise, *p* < 0.05 was considered to be significant.

## Results

### Descriptive Data

After including 135 of the eligible 170 patients attending our CF clinic, thereby achieving the predetermined sample size, further inclusion was ended. Thirty participants required the inhalation of hypertonic saline to induce sputum (=non-expectorating patients), the other 105 were able to expectorate sputum on demand (=expectorating patients). During the study period, 40 patients underwent clinically indicated bronchoscopy (=BAL-group). Because one BAL-sample was lost, the remainder 39 subjects were included in the analyses.

Baseline demographic data are described in Table 2. Fifty-five percent (*n* = 58) of the expectorating patients, 60% (*n* = 18) of the non-expectorating patients and 33.3% (*n* = 13) of the BAL-group were male. Expectorating patients [mean (±*SD*) age: 23.9 (±11.6) years] were significantly older than non-expectorating patients [9.2 (±4.8) years] and patients in the BAL-group [13.4 (±10.3) years] (***p* = 0.000**). Non-expectorating patients had a significantly higher FEV_1_%pred (***p* = 0.000**) and FVC%pred (***p* = 0.004**) compared to expectorating patients and subjects who underwent bronchoscopy.

### Microbiological Analyses

**Figure 1:**
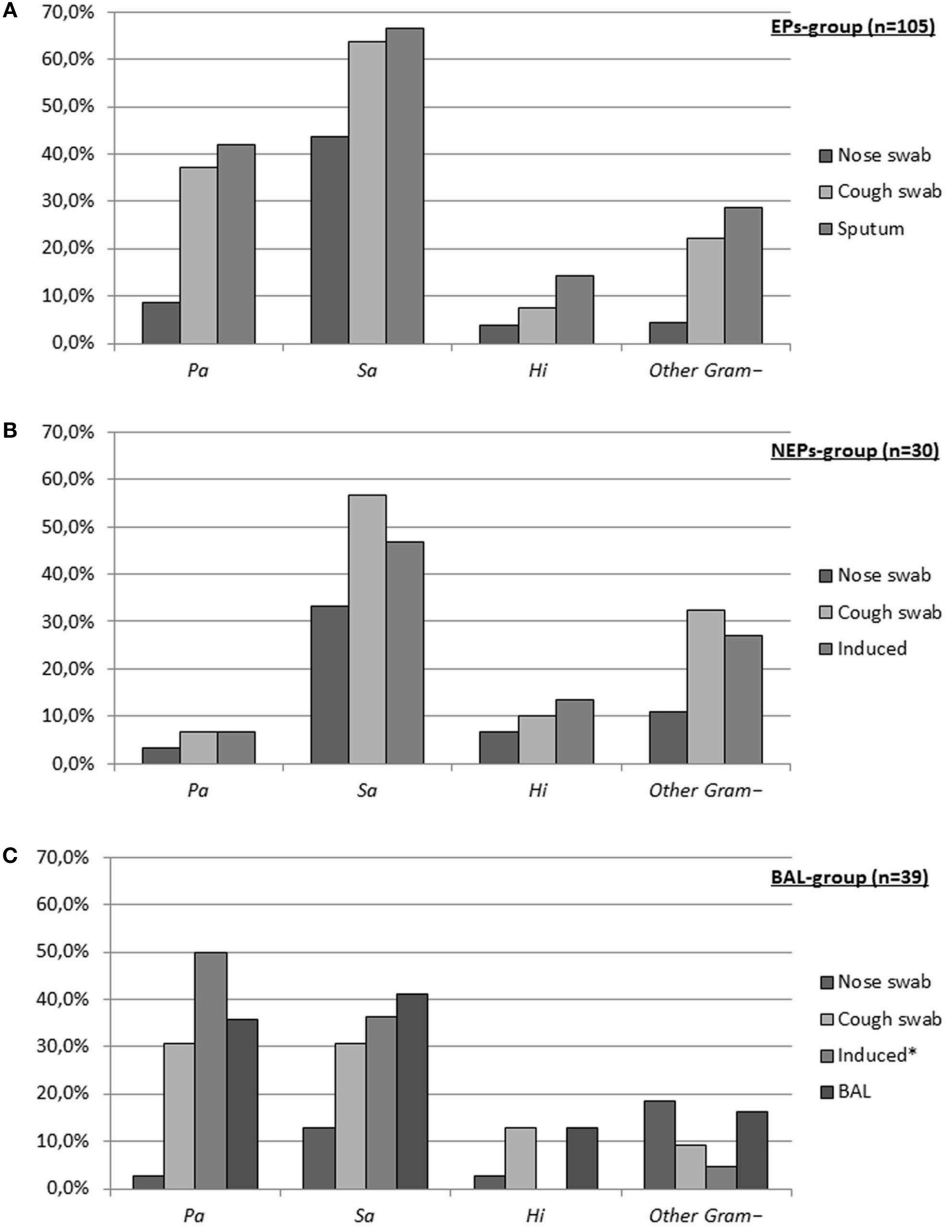
Prevalence of pathogens for each sampling method. **(A)** Expectorating patients (EPs), **(B)** Non-expectorating patients (NEPs), **(C)** BAL-group. (*Pa, Pseudomonas aeruginosa*; *Sa, Staphylococcus aureus* (methicillin-resistant *Sa n* = 1 in expectorating patients and BAL-group and *n* = 0 in non-expectorating patients); *Hi, Haemophilus influenzae*; *Other Gram–*, other gram negative organisms (see Table 3). *Only 22 patients in BAL-group provided induced samples).

In total, 544 respiratory samples were obtained: 174 nasal swabs, 174 cough swabs, 52 induced sputum/cough swabs, 105 spontaneously expectorated sputum samples, and 39 BALs. Figure 1 shows the prevalence of *Pa, Sa, Hi*, and “*other Gram– organisms*” (defined in Table 3) isolated from the respiratory samples in each group.

**Table 3:** Other Gram negative organisms.

**Organism**	**Expectorating patients (*n*)**	**Non-expectorating patients (*n*)**	**BAL-group (*n*)**
*Achromobacter species*	9	1	–
*Achromobacter xylosoxidans*	1	–	–
*Acinetobacter species*	–	2	1
*Bordetella bronchiseptica*	1	–	–
*Burkholderia cenocepacia*	–	1	1
*Burkholderia multivorans*	3	1	1
*Burkholderia vietnamiensis*	1	–	–
*Elizabethkingia species*	1	–	–
*Enterobacter agglomerans complex*	1	–	–
*Enterobacter cloacae complex*	3	1	–
*Escherichia coli*	3	1	–
*Ewingella Americana*	1	–	–
*Hafnia alvei*	1	–	–
*Klebsiella pneumonia*	–	–	2
*Klebsiella oxytoca*	1	2	–
*Moraxella catarrhalis*	1	3	2
*Ochrobactrum anthropic*	–	–	1
*Ochrobactrum species*	1	–	–
*Proteus mirabilis*	4	–	3
*Pseudomonas putida*	–	1	–
*Rhizobium radiobacter*	–	1	2
*Stenotrophomonas maltophilia*	6	3	1
Total *n*	38	17	14

#### Microbiological Prevalence Between Groups

*Pa* was significantly more prevalent in expectorating patients (42.0%) and subjects in the BAL-group (35.9%) compared to non-expectorating patients (6.7%) (***p* = 0.002**). No difference was found between expectorating patients and BAL-subjects (*p* = 0.514; Bonferroni-*p* < 0.017).

*Sa* was detected significantly more in expectorating patients (65.7%) compared to non-expectorating patients (56.7%) and patients who underwent bronchoscopy (41.0%) (***p* = 0.010**). *Sa*-prevalence between non-expectorating patients and BAL-subjects was not significantly different (*p* = 0.662; Bonferroni-*p* < 0.017).

Prevalence of *Hi* and *other Gram– organisms*, was not significantly different between groups (*p* = 0.972; *p* = 0.125, respectively).

#### Microbiological Prevalence Within Groups

##### Expectorating patients (Figure 1A)

Overall microbiological yield of nasal swab, cough swab and expectorated sputum was significantly different between all methods [***p* = 0.000**; nasal swab vs. cough swab (*p* = 0.000^*^); cough swab vs. expectorated sputum (*p* = 0.015^*^); nasal swab vs. expectorated sputum (*p* = 0.000^*^) (^*^Bonferroni-*p* < 0.017)]. Prevalence of *Pa, Sa*, and *other Gram– organisms* in cough swab compared to expectorated sputum was not significantly different [37.1 vs. 42.0% (*p* = 0.049^*^); 63.8 vs. 65.7% (*p* = 0.616^*^); and 22.3 vs. 28.6% (*p* = 0.064^*^) (^*^Bonferroni-*p* < 0.017)], whereas nasal swab yielded these pathogens significantly less compared to cough swab and expectorated sputum [all *p* = 0.000^*^ (^*^Bonferroni-*p* < 0.017)]. *Hi* was more prevalent in expectorated sputum (14.3%) compared to nasal swab (3.8%) and cough swab (7.6%) (***p* = 0.001**). Nasal swab and cough swab yielded *Hi* in the same range (*p* = 0.157; Bonferroni-*p* < 0.017).

##### Non-expectorating patients (Figure 1B)

Overall microbiological yield was significantly different between nasal swab and cough swab (*p* = 0.003^*^; Bonferroni-*p* < 0.017) and a trend toward a significant difference between nasal swab and induced sputum/cough swab was observed (*p* = 0.018; Bonferroni-*p* < 0.017). Overall results for cough swab and induced sputum/cough swab were not significantly different (*p* = 0.542; Bonferroni-*p* < 0.017). Prevalence of *Sa* and *other Gram– organisms* was only different in nasal swab compared to cough swab [33.3 vs. 56.7%; *p* = 0.012^*^ and 10.8 vs. 32.4%; *p* = 0.006^*^ (^*^Bonferroni-*p* < 0.017)]. Separate results for *Pa* and *Hi* could not be calculated as sample sizes were too small (*n* = 2 and *n* = 3, respectively).

##### BAL-group (Figure 1C)

Microbiological yield of cough swab, induced sputum/cough swab and BAL was similar overall, for *Pa, Sa*, and for *other Gram– organisms*. Nasal swab yielded significantly less CF specific pathogens compared to the other methods (all ***p* = 0.000**; Bonferroni-*p* < 0.008). Prevalence of *Hi* was too low (*n* = 6) to compare microbiological yield within the group.

#### Sensitivity, Specificity, and Predictive Values

Sensitivity, specificity, and predictive values are summarized in Table 4.

**Table 4:** Overall sensitivity, specificity, positive, and negative predictive values (PPV and NPV) [95% CI].

**Germ**	**EPs (*****n*** **=** **105)**		**NEPs (*****n*** **=** **30)**		**BAL-Group (*****n*** **=** **39)**		
	**Nose swab**	**Cough swab**	**Nose swab**	**Induced sample**	**Nose swab**	**Cough swab**	**Induced sample^**a**^**
***Pa***	***n*** **=** **44**		***n*** **=** **2**		***n*** **=** **14**		
Sensitivity	20.5% [9.8–35.3]	86.7% [73.2–95.0]	N.A.	N.A.	7.1% [0.2–33.9]	85.7% [57.2–98.2]	100.0% [71.5–100.0]
Specificity	100.0% [94.1–100.0]	98.3% [91.1–100.0]	N.A.	N.A.	100.0% [86.3–100.0]	100.0% [86.3–100.0]	100.0% [71.5–100.0]
PPV	100.0% [–]	97.5% [84.8–99.6]	N.A.	N.A.	100.0% [–]	100.0% [–]	100.0% [–]
NPV	63.5% [60.0–66.9]	90.8% [82.4–95.4]	N.A.	N.A.	65.8% [62.5–69.0]	92.6% [77.6–97.8]	100.0% [–]
***Sa***	***n*** **=** **69**		***n*** **=** **17**		***n*** **=** **16**		
Sensitivity	58.0% [45.5–69.8]	89.9% [80.2–95.8]	47.1% [23.0–72.2]	76.7% [50.1–93.2]	20.0% [4.3–48.1]	75.0% [47.6–92.7]	88.9% [51.8–99.7]
Specificity	83.3% [67.2–93.6]	86.1% [70.5–95.3]	84.6% [54.6–98.1]	92.3% [64.0–99.8]	95.8% [78.9–99.9]	100.0% [85.2–100.0]	100.0% [75.3–100.0]
PPV	87.0% [75.8–93.4]	92.5% [84.6–96.6]	80.0% [50.4–94.0]	92.9% [66.0–98.9]	75.0% [25.5–96.3]	100.0% [–]	100.0% [–]
NPV	50.9% [43.1–58.6]	81.8% [68.4–90.1]	55.0% [42.5–66.9]	75.0% [55.7–87.8]	65.7% [59.5–71.4]	85.2% [71.1–93.1]	92.9% [67.2–98.8]
***Hi***	***n*** **=** **15**		***n*** **=** **3**		***n*** **=** **5**		
Sensitivity	20.0% [4.3–48.1]	40.0% [16.3–67.7]	N.A.	N.A.	20.0% [0.5–71.6]	80.0% [28.4–99.5]	0.0% [0.0–97.5]
Specificity	98.9% [94.0–99.9]	97.8% [92.2–99.7]	N.A.	N.A.	100.0% [89.7–100.0]	97.06% [84.7–99.9]	100.0% [83.9–100.0]
PPV	75.0% [25.0–96.4]	75.0% [40.0–93.1]	N.A.	N.A.	100.0% [–]	80.0% [35.6–96.7]	- [–]
NPV	88.1% [85.2–90.5]	90.7% [86.6–93.7]	N.A.	N.A.	89.5% [84.6–93.0]	97.1% [85.1–99.5]	95.5% [95.5–95.5]
***Other Gram–***	***n*** **=** **38**		***n*** **=** **17**		***n*** **=** **14**		
Sensitivity	6.3% [0.8–20.8]	66.7% [48.2–82.0]	0.0% [0.0–30.9]	80.0% [44.4–97.5]	14.3% [0.4–57.9]	42.9% [9.9–51.6]	66.7% [9.4–99.2]
Specificity	96.2% [89.3–99.2]	94.9% [87.4–98.6]	84.6% [65.1–95.6]	84.6% [65.1–95.6]	80.6% [64.0–91.8]	97.3% [85.8–99.9]	100.0% [84.6–100.0]
PPV	40.0% [10.5–79.2]	84.6% [67.3–93.6]	– [–]	66.7% [43.5–83.8]	12.5% [2.0–49.7]	75.0% [26.6–96.1]	100.0% [–]
NPV	71.7% [69.6–73.7]	87.1% [80.6–91.6]	68.8% [65.1–72.2]	91.7% [75.9–97.5]	82.9% [77.4–87.2]	90.0% [82.5–94.5]	95.7% [81.6–99.1]

*EPs, expectorating patients; NEPs, non-expectorating patients; BAL-group, patients requiring clinically indicated bronchoscopy*.

a *Only 22 induced sputum samples. Pa, Pseudomonas aeruginosa; N.A., not applicable (because of low sample size); Sa, Staphylococcus aureus; Hi, Haemophilus influenzae; Other Gram–, other gram negative organisms (see Table 3)*.

Nasal swab had low sensitivity (expectorating patients: 6.3–58.0%; non-expectorating patients: 0.0–100.0%; and BAL-group: 7.1–20.0%) and high specificity (expectorating patients: 83.3–100.0%; non-expectorating patients: 84.6–100.0%; and BAL-group: 80.6–100.0%) depending on the pathogen in all three patient groups compared to the reference sample (expectorated sputum, cough swab and BAL, respectively). In addition, wide ranging predictive values were observed in all three patient groups.

##### Expectorating patients

Sensitivity (86.7–89.9%) and specificity (86.1–98.3%) results of the cough swab were high for all pathogens, except the sensitivity for *Hi* (40.0%) and *other Gram– organisms* (66.7%). Likewise, all predictive values were high for all pathogens (PPV:75.0–100.0%; and NPV:81.6–90.8%), except the PPV of *other Gram– organisms* (40.0%).

##### Non-expectorating patients

In non-expectorating patients, sensitivity and specificity of induced sputum/cough swab were 76.7 and 92.3% for *Sa* and 80.0 and 84.6% for *other Gram– organisms*. PPV and NPV were high (75.0–92.9%), except the PPV of *other Gram– organisms* (66.7%). Results could not be calculated for *Pa* and *Hi* as prevalence of these pathogens was low (*n* = 2 and *n* = 3, respectively).

##### BAL-group

Results for sensitivity, specificity and predictive values were all high (>75.0%) and in the same range for cough swab and induced sputum/cough swab compared to BAL, except the sensitivity of *Hi* (80.0% in cough swab vs. 0.0% in induced sputum/cough swab) and the sensitivity of *other Gram– organisms* (42.9% in cough swab vs. 66.7% in induced sputum/cough swab).

## Discussion

The present study investigated the accuracy of several respiratory sampling techniques for bacterial culture in patients with cystic fibrosis. In expectorating patients we examined nasal swab, cough swab and spontaneously expectorated sputum. Nasal swab and induced sputum were compared to cough swab in non-expectorating subjects. In patients requiring a clinically indicated bronchoscopy, microbiological yield of nasal swab, cough swab, and induced sputum/cough swab was compared to the result of the bronchoalveolar lavage. Eventually, we wanted to find out whether the use of a cough swab is of clinical value in non-expectorating patients.

Sensitivity, specificity, and predictive values revealed that a nasal swab was not appropriate to detect pathogens present in the lower airways in all three patient groups (Table 4), which is also reflected in the prevalence chart (Figure 1). This finding confirms a previous study comparing nasal swab, throat swab, and sputum. A substantial difference was observed between nose microbiota on the one hand and microbiota from the throat and sputum on the other hand (15). A more recent study comparing 25 sets of nasopharyngeal, oropharyngeal (OP), and bronchoalveolar lavage suggested that the lungs of infants with CF have indeed their own microbiome which seems like, but is not identical to, the upper respiratory tract (16). Likewise, Taylor et al. compared nasal suctioning to cough swab (17). Although an equal prevalence of the most common bacteria (*Pa, Sa, Hi*) was reported with both sampling techniques, the authors suggested that nasopharyngeal suctioning is not routinely warranted, because of the lack of benefit over throat swabs in detection of CF pathogens.

Despite a significantly different overall prevalence of pathogens in cough swab compared to sputum in expectorating patients, we did not find a significant difference when specifying for prevalence of *Pa* or *Sa*. On the other hand, the high PPV and NPV for *Pa* and *Sa* are a positive finding. In other words, when *Pa* or *Sa* were detected in the cough swab of expectorating patients, the pathogen was most likely also isolated in the concomitantly obtained spontaneously expectorated sputum. Although avoiding oropharyngeal contamination, it is possible that expectorating patients coughed sputum onto the cough swab, thereby giving a higher quality specimen compared to non-expectorating patients. Therefore, caution is warranted when extrapolating these results to the non-expectorating patients. Likewise, the findings in the BAL-group should be read carefully. Microbiological yield of cough swab and induced sputum was similar to that of a BAL and was reflected by the high sensitivity, specificity, and predictive values. These results are confirmed by some authors (8–10, 18, 19), but partially refuted by others (7, 9, 11, 18–21). Indeed, Ramsey et al. found a high PPV of an OP culture yielding *Pa* (83%) or *Sa* (91%) compared to BAL. However, they also report lower NPVs (<80%) as a result of a high frequency of false negatives. It is suggested that OP cultures yielding *Pa* and *Sa* are highly predictive of the presence of these pathogens in the lower airways of patients with CF, but a negative culture does not rule out their presence (8). This statement was supported by another study which reported a PPV of 100% and NPV of 21% for *Pa* (10). Contrary, other studies report low PPVs (41–69%) and high NPVs (85–98%) for OP cultures, meaning a negative culture is likely to rule out, but a positive culture does not “rule in” lower airway infection (9, 18, 19, 21).

Jung et al. evaluated throat swabs, spontaneous expectorated sputum and BAL from stable patients with CF for the detection of *Pa* (7). As confirmed by our results, sputum samples were found of equal value as samples obtained through bronchoscopy to detect *Pa* colonization. However, throat swabs were not suitable for characterizing bacterial conditions in participants' lower airways. A possible explanation is that cough swabs, as performed in our study, are less prone to upper airway contamination than throat swabs as used by Jung. In addition, our cough swab was obtained after a short airway clearance session, improving mucociliary clearance, and the upward movement of pathogens from the lower airways. It has been shown that the sensitivity of a throat swab after physiotherapy is higher compared to a normal throat swab (82–100 vs. 40–57%) (22). Also, our patients required bronchoscopy on a clinical basis, unlike the patients in Jung's study. It has been demonstrated that yield and adequacy of samples is significantly higher in symptomatic children compared to those who are clinically stable (23), which might have influenced results in the BAL-group.

Studies assessing the clinical value of pre- and post- induction obtained sputum samples (after inhalation of HS) in non-expectorating patients, demonstrated an improved pathogen detection in the latter, which led to changes in patient management (11, 12, 20, 23–27). Additionally, a good bacteriologic correlation has been demonstrated between induced sputum samples and BAL in two smaller groups of symptomatic (*n* = 35) (12) and mixed (symptomatic and asymptomatic) patients with CF (*n* = 10) (28) and is confirmed by our results in the BAL-group. It has been suggested that in symptomatic patients sputum induction will correctly identify pathogens in the lower airways in most of these patients and should be performed and handled accordingly prior to bronchoscopy tailored treatment (12). On the other hand, an Australian group found that induced sputum compared to BAL, although not necessarily taken on the same day, in a larger group of patients with CF (*n* = 61) was not highly sensitive (36.8%) or specific (69.0%), with (50 and 60.9%, respectively) or without (27.3 and 78.9%, respectively) airway clearance (29). Therefore, it was concluded that the use of induced sputum should not be recommended in routine clinical practice (29). In addition, a recent study demonstrated a bactericidal effect of HS on *Pa* (30). As *Pa* is a typical CF-pathogen which is associated with reduced lung function and shortened life-expectancy in patients with CF (31), it is crucial to accurately detect an infection with this pathogen as early as possible.

The present study has several limitations. First, false-positive BAL cultures could be present among the results. Although the oral route was used to insert the bronchoscope and suctioning was avoided prior to wedging the scope in one lobe or the other, contamination by upper airway flora might have occurred and could have been avoided better by using a laryngeal mask. Secondly, BAL was not used as “gold standard” in all three patient groups. However, it should be noted that BAL has some limitations itself. Indeed, no consensus exists on how BAL should be performed, i.e., single-lobe, two-lobe, or comprehensive six-lobe BAL. Current guidelines recommend two-lobe BAL for children with CF (32). On the other hand, it has been demonstrated that six-lobe BAL is safe, well-tolerated and superior to single-lobe and two-lobe BAL, suggesting that bacterial communities might be heterogeneously spread throughout the airways (33). A most recent study wanted to account for “false” false positive results when comparing other sampling methods to BAL (12). Therefore, a “combined gold standard” was used, consisting of all pathogens identified by sputum induction and six-lobe BAL, as they presumed that pathogens isolated in sputum induction alone and not in BAL were rather additional lower airway pathogens instead of false positives. By using this “combined gold standard,” six-lobe BAL and induced sputum alone were found to have a sensitivity of 81 and 63%, respectively (12). This questions whether the use of BAL alone as “gold standard” is adequate when calculating sensitivity and specificity (7–10, 19, 21, 29), even when it is six-lobe BAL as was performed in our study. Thirdly, although in the same range of other similar studies (26–28), sample size of non-expectorating patients is quite small, possibly resulting in a lower prevalence of some typical CF-pathogens. However, the low prevalence of *Pa* (*n* = 2) and *Hi* (*n* = 3) should not be attributed to younger age of non-expectorating patients compared to that of expectorating patients. Indeed, subjects in the BAL-group were also significantly younger than expectorating patients, but demonstrated similar rates of positive cultures. It should more likely be attributed to the fact that expectorating patients and patients in the BAL-group showed more signs of disease, reflected by a lower lung function, and thus, are more likely to be colonized with disease causing CF-pathogens. It has been demonstrated that yield and adequacy of samples is significantly higher in symptomatic children compared to those who are clinically stable (24). Fourthly, not all patients in the BAL-group (22 out of 39) provided an induced sample. This implies that prevalence of pathogens in induced samples might have been falsely higher than it really was. However, we found concordance in results of cough swab, induced sputum and BAL in these 22 patients. Lastly, if a patient was unable to expectorate sputum after HS-inhalation, we performed a second cough swab instead of OP suctioning because children do not tolerate OP suctioning very well (21, 29). Therefore, it is suggested that clinicians should consider patient age and the risk of increased anxiety if limited sensitivity of a sampling technique has been demonstrated (29).

With this study, we could not disprove the existing controversy with regard to respiratory culture sampling in the clinically stable non-expectorating patient with CF. However, for now, we will not change our current practice of using cough swab for bacterial culture to another sampling method in this patient group, because: (I) our results did not reveal a difference between cough swab and induced samples, whether or not compared to BAL, in both our non-expectorating patients (no results for *Pa* and *Hi* due to low prevalence) and BAL-group (except sensitivity for *Hi*); (II) of the limited repeatability of bronchoscopy (7); (III) of the limited tolerability of OP suctioning, especially in children (29); (IV) of the known anti-*Pa* effect of HS which is used with sputum induction (30); and (V) sputum induction is time-consuming (20–45 min) and expensive (24, 28). Nonetheless, in symptomatic patients, sputum induction should be performed prior to BAL (12). Additionally, despite the fact that our non-expectorating patients preferred the nasal swab as sampling method (34), we will not use nasal swab as sampling method because our results demonstrated that it was not appropriate to detect pathogens of the lower airways. Likewise, we will obtain spontaneously expectorated sputum from patients who are able to expectorate, because of the overall significant superiority compared to cough swab. In addition, this sampling technique was preferred over cough swab and nasal swab in expectorating patients (34).

Nevertheless, respiratory sampling in non-expectorating patients remains challenging. Therefore, it is important to continue the search for the best sampling technique that is repeatable, which is currently not the case for BAL. A study comparing microbiological yield and sensitivity, specificity and predictive values of normal cough swab, induced cough swab and induced OP suctioning to BAL in these non-expectorating patients will be of great interest. In addition, it might be considered to include non-expectorating patients suffering from other chronic respiratory diseases, such as primary ciliary dyskinesia (PCD) and non-CF bronchiectasis. Like in patients with CF, these patients require close surveillance in regard to respiratory bacterial growth (35–37). Typical CF-pathogens, such as *Pa, Sa, Hi*, and *Streptococcus pneumoniae*, are also predominant in the culture growths of patients with PCD and non-CF bronchiectasis. Furthermore, presence of *Pa* is associated with greater impairment in lung function, increased airway inflammation, more frequent exacerbations, worse quality of life, greater risk of hospitalization, and increased mortality (35–37). Treatment decisions, including the selection of antibiotics, are often tailored toward culture history and microbial sensitivity (37). Therefore, patients with PCD and non-CF bronchiectasis will also benefit from improved sampling techniques.

## Conclusion

The present study investigated the accuracy of several respiratory sampling techniques for bacterial culture in patients with cystic fibrosis. Because of the lower microbiological yield, low sensitivity and the wide range in predictive values of nasal swab compared to the other methods, we conclude that nasal swab is not appropriate to detect pathogens present in the lower airways of these patients. Cough swab and induced sputum showed similar results for microbiological yield, specificity, sensitivity, and predictive values, both in non-expectorating patients (however, no results for *Pa* and *Hi*) and patients undergoing clinically indicated bronchoscopy. Additionally, cough swab and induced sputum results were also similar to BAL, except for *Hi* (induced samples) and *other Gram– organisms* (both cough swab and induced samples). Our findings suggest that cough swab might be helpful in detecting the presence of some typical CF pathogens in the lower airways of clinically stable patients with CF. However, in symptomatic patients who are unable to expectorate and who have a negative cough swab and induced sample, BAL should be performed as it currently remains the “gold standard.”

## Data Availability Statement

Data may compromise the privacy of study participants and may not be shared publicly. Data are available upon request to the corresponding author (HE).

## Author Contributions

HE drafted the manuscript. DP and LE completed the methods section (microbiological analyses). ED and AM completed the methods section (bronchoscopy protocol). DP, ED, LE, PV, and AM provided critical input to the rest of the manuscript, and all the authors approved the final version.

### Conflict of Interest Statement

The authors declare that the research was conducted in the absence of any commercial or financial relationships that could be construed as a potential conflict of interest.

## References

[1] Cohen-Cymberknoh M Shoseyov D Kerem E. Managing cystic fibrosis: strategies that increase life expectancy and improve quality of life. Am J Respir Crit Care Med. (2011) 183:1463–71. 10.1164/rccm.201009-1478CI 21330455

[2] Smyth AR Bell SC Bojcin S Bryon M Duff A Flume P. European cystic fibrosis society standards of care: best practice guidelines. J Cyst Fibros. (2014) 13:S23–42. 10.1016/j.jcf.2014.03.010 24856775

[3] Gaspar MC Couet W Olivier JC Pais AACC Sousa JJS. *Pseudomonas aeruginosa* infection in cystic fibrosis lung disease and new perspectives of treatment: a review. Eur J Clin Microbiol Infect Dis. (2013) 32:1231–52. 10.1007/s10096-013-1876-y 23619573

[4] Clancy JP Jain M. Personalized medicine in cystic fibrosis: dawning of a new era. Am J Respir Crit Care Med. (2012) 186:593–7. 10.1164/rccm.201204-0785PP 22723294

[5] Lahiri T Hempstead SE Brady C Cannon CL Clark K Condren ME. Clinical practice guidelines from the cystic fibrosis foundation for preschoolers with cystic fibrosis. Pediatrics (2016) 137:e20151784. 10.1542/peds.2015-1784 27009033

[6] Connett GJ. Bronchoalveolar lavage. Paediatr Respir Rev. (2000) 1:52–6. 10.1053/prrv.2000.0007 16263445

[7] Jung A Kleinau I Schönian G Bauernfeind A Chen C Griese M. Sequential genotyping of *Pseudomonas aeruginosa* from upper and lower airways of cystic fibrosis patients. Eur Respir J. (2002) 20:1457–63. 10.1183/09031936.02.00268002 12503704

[8] Ramsey BW Wentz KR Smith AL Richardson M Williams-Warren J Hedges DL. Predictive value of oropharyngeal cultures for identifying lower airway bacteria in cystic fibrosis patients. Am Rev Respir Dis. (1991) 144:331–7. 10.1164/ajrccm/144.2.331 1859056

[9] Rosenfeld M Emerson J Accurso F Armstrong D Castile R Grimwood K. Diagnostic accuracy of oropharyngeal cultures in infants and young children with cystic fibrosis. Pediatr Pulmonol. (1999) 28:321–8. 10.1002/(SICI)1099-0496(199911)28:5<321::AID-PPUL3>3.0.CO;2-V 10536062

[10] Equi AC Pike SE Davies J Bush A. Use of cough swabs in a cystic fibrosis clinic. Arch Dis Child. (2001) 85:438–9. 10.1136/adc.85.5.438 11668115 PMC1718986

[11] Ho SA Ball R Morrison LJ Brownlee KG Conway SP. Clinical value of obtaining sputum and cough swab samples following inhaled hypertonic saline in children with cystic fibrosis. Pediatr Pulmonol. (2004) 38:82–7. 10.1002/ppul.20035 15170878

[12] Ronchetti K Tame JD Paisey C Thia LP Doull I Howe R. The CF-Sputum Induction Trial (CF-SPIT) to assess lower airway bacterial sampling in young children with cystic fibrosis: a prospective internally controlled interventional trial. Lancet Respir Med. (2018) 6:461–71. 10.1016/S2213-2600(18)30171-1 29778403 PMC5971213

[13] Garcia L Respiratory tract cultures (Chapter 3.11). In: Garcia L editor. Clinical Microbiology Procedures Handbook, 3rd ed. Washington, DC: ASM Press (2010). p. 321–409.

[14] Parikh R Mathai A Parikh S Sekhar GC Thomas R. Understanding and using sensitivity, specificity and predictive values. Indian J Opthalmol. (2008) 56:45–50. 10.4103/0301-4738.37595 18158403 PMC2636062

[15] Boutin S Graeber SY Weitnauer M Panitz J Stahl M Clausznitzer D. Comparison of microbiomes from different niches of upper and lower airways in children and adolescents with cystic fibrosis. PLoS ONE (2015) 10:e0116029. 10.1371/journal.pone.0116029 25629612 PMC4309611

[16] Prevaes SM de Steenhuijsen Piters WA de Winter-de Groot KM Janssens HM Tramps-Stranders GA Chu ML. Concordance between upper and lower airway microbiota in infants with cystic fibrosis. Eur Respir J. (2017) 49:1602235. 10.1183/13993003.02235-2016 28356374

[17] Taylor L Corey M Matlow A Sweezey NB Ratjen F. Comparison of throat swabs and nasopharyngeal suction specimens in non-sputum producing patients with cystic fibrosis. Pediatr Pulmonol. (2006) 41:839–43. 10.1002/ppul.20451 16850448

[18] Burns JL Gibson RL McNamara S Yim D Emerson J Rosenfeld M. Longitudinal assessment of *Pseudomonas aeruginosa* in young children with cystic fibrosis. J Infect Dis. (2001) 183:444–52. 10.1086/318075 11133376

[19] Armstrong DS Grimwood K Carlin JB Carzino R Olinsky A Phelan PD. Bronchoalveolar lavage or oropharyngeal cultures to identify lower respiratory pathogens in infants with cystic fibrosis. Pediatr Pulmonol. (1996) 21:267–75. 10.1002/(SICI)1099-0496(199605)21:5<267::AID-PPUL1>3.0.CO;2-K 8726151

[20] Hoppe JE Towler EE Wagner BD Accurso FJ Sagel SD Zemanick ET. Sputum induction improves detection of pathogens in children with cystic fibrosis. Pediatr Pulmonol. (2015) 50:638–46. 10.1002/ppul.23150 25565628 PMC4495008

[21] Doumit M Belessis Y Stelzer-Braid S Mallitt KA Rawlinson W Jaffe A. Diagnostic accuracy and distress associated with oropharyngeal suction in cystic fibrosis. J Cyst Fibros. (2016) 15:473–8. 10.1016/j.jcf.2015.09.001 26388518

[22] Kabra SK Alok A Kapil A Aggarwal G Kabra M Lodha R. Can throat swab after physiotherapy replace sputum for identification of microbial pathogens in children with cystic fibrosis? Indian J Pediatr. (2004) 71:21–3. 10.1007/BF02725650 14979380

[23] Zampoli M Pillay K Carrara H Zar HJ Morrow B. Microbiological yield from induced sputum compared to oropharyngeal swab in young children with cystic fibrosis. J Cyst Fibros. (2016) 15:605–10. 10.1016/j.jcf.2016.01.001 26825010

[24] Al-Saleh S Dell SD Grasemann H Yau YCW Waters V Martin S. Sputum induction in routine clinical care of children with cystic fibrosis. J Pediatr. (2010) 157:1006–11. 10.1016/j.jpeds.2010.06.001 20630539

[25] Zemanick ET Wagner BD Robertson CE Stevens MJ Szefler SJ Accurso FJ. Assessment of airway microbiota and inflammation in cystic fibrosis using multiple sampling methods. Ann Am Thorac Soc. (2015) 12:211–9. 10.1513/AnnalsATS.201407-310OC 25474078 PMC4342834

[26] Aaron SD Kottachchi D Ferris WJ Vandemheen KL St Denis ML Plouffe A. Sputum versus bronchoscopy for diagnosis of *Pseudomonas aeruginosa* biofilms in cystic fibrosis. Eur Respir J. (2004) 24:631–7. 10.1183/09031936.04.00049104 15459143

[27] Henig NR Tonelli MR Pier MV Burns JL Aitken ML. Sputum induction as a research tool for sampling the airways of subjects with cystic fibrosis. Thorax (2001) 56:306–11. 10.1136/thorax.56.4.306 11254823 PMC1746031

[28] Blau H Linnane B Carzino R Tannenbaum E-L Skoric B Robinson PJ. Induced sputum compared to bronchoalveolar lavage in young, non-expectorating cystic fibrosis children. J Cyst Fibros. (2014) 13:106–10. 10.1016/j.jcf.2013.05.013 23806622

[29] D'Sylva P Caudri D Shaw N Turkovic L Douglas T Bew J. Induced sputum to detect lung pathogens in young children with cystic fibrosis. Pediatr Pulmonol. (2017) 52:182–9. 10.1002/ppul.23636 27905200

[30] Michon AL Jumas-Bilak E Chiron R Lamy B Marchandin H. Advances toward the elucidation of hypertonic saline effects on *Pseudomonas aeruginosa* from cystic fibrosis patients. PLoS ONE (2014) 9:e90164. 10.1371/journal.pone.0090164 24587256 PMC3938589

[31] Emerson J Rosenfeld M McNamara S Ramsey BW Gibson RL. *Pseudomonas aeruginosa* and other predictors of mortality and morbidity in young children with cystic fibrosis. Pediatr Pulmonol. (2002) 34:91–100. 10.1002/ppul.10127 12112774

[32] Brennan S Gangell C Wainwright C Sly PD. Disease surveillance using bronchoalveolar lavage. Paediatr Respir Rev. (2008) 9:151–9. 10.1016/j.prrv.2008.01.002 18694706

[33] Gilchrist FJ Salamat S Clayton S Peach J Alexander J Lenney W. Bronchoalveolar lavage in children with cystic fibrosis: how many lobes should be sampled? Arch Dis Child. (2011) 96:215–7. 10.1136/adc.2009.177618 20930010

[34] Eyns H De Wachter E Malfroot A Vaes P. Acute pain perception during different sampling methods for respiratory culture in cystic fibrosis patients. J Pain Symptom Manage. (2018) 55:872–80. 10.1016/j.jpainsymman.2017.11.004 29154891

[35] Mirra V Werner C Santamaria F. Primary Ciliary Dyskinesia: an update on clinical aspects, genetics, diagnosis, and future treatment strategies. Front Pediatr. (2017) 5:135. 10.3389/fped.2017.00135 28649564 PMC5465251

[36] Pizzutto SJ Hare KM Upham JW. Bronchiectasis in children: current concepts in immunology and microbiology. Front Pediatr. (2017) 5:123. 10.3389/fped.2017.00123 28611970 PMC5447051

[37] Polineni D Davis SD Dell SD. Treatment recommendations in primary ciliary dyskinesia. Pediatr Respir Rev. (2016) 18:39–45. 10.1016/j.prrv.2015.10.002 26586601

